# Host-Dependent Producibility of Recombinant *Cypridina noctiluca* Luciferase With Glycosylation Defects

**DOI:** 10.3389/fbioe.2022.774786

**Published:** 2022-02-07

**Authors:** Yasuo Mitani, Rie Yasuno, Kiyohito Kihira, KwiMi Chung, Nobutaka Mitsuda, Shusei Kanie, Azusa Tomioka, Hiroyuki Kaji, Yoshihiro Ohmiya

**Affiliations:** ^1^ Bioproduction Research Institute, National Institute of Advanced Industrial Science and Technology (AIST), Sapporo, Japan; ^2^ Cellular and Molecular Biotechnology Research Institute, AIST, Tsukuba, Japan; ^3^ Japan Aerospace Exploration Agency (JAXA), Tsukuba, Japan; ^4^ Bioproduction Research Institute, AIST, Tsukuba, Japan; ^5^ Biomedical Research Institute, AIST, Ikeda, Japan; ^6^ Osaka Institute of Technology (OIT), Osaka, Japan

**Keywords:** luciferase, *Cypridina noctiluca*, glycosylation, recombinant protein expression, mass spectrometry

## Abstract

*Cypridina noctiluca* luciferase (CLuc) is a secreted luminescent protein that reacts with its substrate (Cypridina luciferin) to emit light. CLuc is known to be a thermostable protein and has been used for various research applications, including *in vivo* imaging and high-throughput reporter assays. Previously, we produced a large amount of recombinant CLuc for crystallographic analysis. However, this recombinant protein did not crystallize, probably due to heterogeneous N-glycan modifications. In this study, we produced recombinant CLuc without glycan modifications by introducing mutations at the N-glycan modification residues using mammalian Expi293F cells, silkworms, and tobacco Bright Yellow-2 cells. Interestingly, recombinant CLuc production depended heavily on the expression hosts. Among these selected hosts, we found that Expi293F cells efficiently produced the recombinant mutant CLuc without significant effects on its luciferase activity. We confirmed the lack of N-glycan modifications for this mutant protein by mass spectrometry analysis but found slight O-glycan modifications that we estimated were about 2% of the ion chromatogram peak area for the detected peptide fragments. Moreover, by using CLuc deletion mutants during the investigation of O-glycan modifications, we identified amino acid residues important to the luciferase activity of CLuc. Our results provide invaluable information related to CLuc function and pave the way for its crystallographic analysis.

## Introduction

Luciferase is an enzyme that oxidizes the substrate luciferin; some are cellular proteins, while others are secreted. Secreted luciferases have been reported in a wide range of phyla including Arthropoda, Mollusca, and Annelida (Shimomura 2012). Among them, luciferase genes have been cloned from Copepoda *Gaussia princeps* (Bryan and Szent-Gyorgyi 2001) and *Metridia longa* (Markova et al., 2004); from Ostracoda *Vargula hilgendorfii* (Thompson et al., 1989), *Cypridina noctiluca* (Nakajima et al., 2004), and Caribbean species (Hensley et al., 2021); from the deep-sea shrimp *Oplophorus gracilirostris* (Inouye et al., 2000); and from syllid worms like *Odontosyllis undecimdonta* (Mitani et al., 2018; Schultz et al., 2018) and related species (Mitani et al., 2019). Secreted luciferases are useful for high-throughput analysis because the assay is made less difficult by using culture supernatants. Thus far, luciferases from the genera *Oplophorus* and *Gaussia* have been widely used in application research (Hall et al., 2012; Suzuki and Inouye 2014; Markova et al., 2019). *Oplophorus* luciferase (OLuc) has some advantages, such as its catalytic domain with low molecular weight, excellent heat stability, and relatively easy expression in both prokaryotic and eukaryotic cells when some amino acid mutations are incorporated (Hall et al., 2012; Inouye et al., 2013). In the case of *Gaussia* luciferase (GLuc), a production method for low-temperature expression in the presence of chaperones has been established in an *Escherichia coli* expression system (Inouye and Sahara 2008). The three-dimensional (3D) structures of these luciferases have been elucidated by crystallization and X-ray diffraction of the partial domain exhibiting the catalytic function in OLuc (Tomabechi et al., 2016) and by heteronuclear NMR for GLuc (Wu et al., 2020).

*C. noctiluca* luciferase (CLuc) has been developed for applied research by modifying its surface with fluorescent molecules to emit infrared light (Wu et al., 2009) and by optimizing it for a high-throughput reporter assay (Tochigi et al., 2010). CLuc is also a stable protein with high thermal stability and has high potential for further applications. However, CLuc has a higher molecular weight (62–68 kDa) than OLuc and GLuc, and may have up to 17 potential disulfide bonds between the 34 cysteine residues in its 553 amino acids, while GLuc has only 5 disulfide bonds (Wu et al., 2020), thus making protein production and applied research with CLuc difficult (Inouye and Sahara 2008). There are no reports on the prokaryotic expression of full-length CLuc. Although CLuc does not show homology to other luciferases, its first- and second-half regions display weak homology to the von Willebrand factor type D (VWD) domain (Oakley 2005). These two VWD-like domains show approximately 20% homology to each other. The first half, containing 302 amino acids, has been expressed using an *E. coli* system, resulting in 45% activity of the intact protein (Hunt et al., 2017). However, there are no biochemical reports on the residues directly involved in CLuc activity, and there is no information on its 3D structure. The establishment of a system that can stably and massively produce CLuc is expected to lead to further progress in crystallographic analysis and future applications. Structural information will also help to modify CLuc using a simple approach to bioconjugation (Hu et al., 2018).

Secreted proteins in addition to luciferases need to undergo appropriate folding, including disulfide bond formation during the secretory process, and most secreted proteins undergo post-translational modification such as glycosylation (Helenius and Aebi 2001). Although glycosylation is generally considered to contribute to protein stability by increasing folding efficiency (Helenius and Aebi 2001; Wang et al., 2018), its effects on protein function itself are inconsistent. For example, it was reported that some hormones maintain their ability to bind to receptors when glycosylation is removed by hydrogen fluoride (HF) treatment, but the downstream signals are not transduced (Lapthorn et al., 1994). In another case, human follicle-stimulating hormone (hFSH) remains fully active even after glycan chains are removed *via* point mutation (Fox et al., 2001); the folding of these glycosylation-deficit mutants is unaffected (Wilbers et al., 2016). Since glycosylation can prevent crystallization due to its heterogeneity (Lapthorn et al., 1994; Columbus 2015), glycans can be removed by deglycosylation enzyme treatment or mutagenesis to the modification site (Woods 2018). CLuc is the only secreted luciferase whose glycan structure has been analyzed and reported to remain fully active even after enzymatic removal of the glycan from wild-type (Wt) recombinant protein produced in suspension-cultured tobacco Bright Yellow-2 (BY-2) cells (Mitani et al., 2017). In the case of mutant CLuc lacking glycan modification sites expressed in COS1 cells, its specific activity has been reported to be reduced to approximately 20% when compared to the Wt recombinant protein produced in COS1 cells. However, due to the low productivity, quantitative analysis using purified CLuc proteins remains to be conducted (Yasuno et al., 2018).

In this study, we tried to produce mutant recombinant CLuc lacking N-glycan modification in available hosts in our laboratory, including Expi293F cells, silkworms, and BY-2 cells. CLuc has two conserved N-glycan modification motifs, NIT and NTS, in its amino acid sequence, starting at residues N182 and N404, respectively. These two motifs were mutated to produce a double mutant recombinant protein (Dmt CLuc) containing T184A + N404D substitutions. In comparing the specific activities of the obtained recombinant CLuc by measuring the protein amount using highly purified CLuc or anti-CLuc antibody, we found that the mutant CLuc produced by Expi293F cells showed almost the same specific activity as the Wt. We also found important amino acid residues involved in the luciferase activity by expressing amino-terminal serial deletion mutants of Dmt CLuc.

## Materials and Methods

### Recombinant Proteins Used in This Study

In this study, we produced Wt and Dmt CLuc using Expi293F cells, silkworms, and BY-2 cells, designating the recombinant proteins CLuc_EX_, CLuc_SW_, and CLuc_BY_, respectively. For clarity, we designated Dmt CLuc previously expressed using COS1 cells (Yasuno et al., 2018) as Dmt CLuc_CO_ in this study.

### *Cypridina noctiluca* Luciferase Expression Using Expi293 Expression System

Expression vectors for recombinant Wt CLuc_EX_ and Dmt CLuc_EX_, pcDNA-CL (Nakajima et al., 2004), and pcDNA-CL (T184A + N404D) (Yasuno et al., 2018) were prepared as previously described. The pcDNA-CL (T184A + N404D) was modified by the insertion of the sequence for His-tag and TEV protease (HHHHHHENLYFQG) following the predicted signal sequence (1–18 a.a.). Expression vectors for deletion mutants were designed with deleted amino-terminal or carboxyl-terminal residues of the mature region by modification of the His-TEV-pcDNA-CL (T184A + N404D) plasmid. These insertion and deletion modifications were outsourced to Genscript Japan. Each expression vector was transfected into Expi293F mammalian cells (Thermo Fisher Scientific, MA, United States) according to the manufacturer’s instructions. On day 5 post-transfection, the culture medium was centrifuged at 350 × g for 10 min, and the resulting supernatant was used for the following analysis, hereafter called the medium fraction. Precipitated cells were lysed with 10 mM Tris-HCl (pH 7.4) by sonication, the cell lysate was centrifuged at 20,000 × g for 5 min, and the resulting supernatant was used as the cell extract fraction. Immunoblot analysis for CLuc proteins was conducted as described previously (Yasuno et al., 2018), except that 10 μL of each medium fraction sample was prepared under reducing conditions with dithiothreitol before electrophoresis and that the anti-CLuc antibody was raised using purified recombinant CLuc in rabbit. The activities of the recombinant CLuc proteins were measured as described previously (Yasuno et al., 2018), and the intensities were obtained as relative light units (RLUs).

### LC-MS/MS Analyses of *Cypridina noctiluca* Luciferase Mutated at N-Glycosylation Sites

Dmt CLuc_EX_ was S-reduced and alkylated as described previously (Mitani et al., 2017). The protein was digested with a mixture of trypsin and Lys-C endopeptidase and/or chymotrypsin (TL/C/CTL). An aliquot of each digest was heated at 80°C for 2 h in 0.1% TFA to remove sialic acid and to increase the detection sensitivity of glycopeptides. Each digest was analyzed by an LC-MS system using a nanoflow LC (Ultimate 3000, Thermo Fisher Scientific) and Orbitrap Fusion Tribrid mass spectrometer (Thermo Fisher Scientific). MS and MS/MS spectra were obtained by the Orbitrap analyzer with a resolution of 120,000 at 200 m/z. Data dependently selected precursor ions were fragmented by high-energy collision-induced dissociation (HCD) and HCD-fragment-triggered EThcD. Glycan peptide cluster analysis was performed by glycan-heterogeneity-based relational identification of glycopeptide signals on elution profile (Glyco-RIDGE; Togayachi et al., 2018). MS/MS spectra were searched using Mascot (ver. 2.5.1, Matrix Science, MA, United States) with the UniProt protein sequence database for *Homo sapiens* including sequences of CLuc (gi|41152712) with mutations (T184A + N404D). Search parameters were set as described previously (Mitani et al., 2017) with slight modifications; Hex(1) HexNAc(1)_Nli(ST) or Hex(1) HexNAc(1) NeuAc(2)_Nli(ST); Nli: neutral loss and ignore mass were set, enzyme; semi for each enzyme, and maximum missed cleavage for chymotrypsin; 6.

### *Cypridina noctiluca* Luciferase Expression in Silkworm and Initial Purification

Recombinant CLuc_SW_ proteins were produced using the silkworm expression system as described previously (Maeda et al., 1985). CLuc ORF was amplified by PCR and inserted into the pHS02 vector under the control of a polyhedron promoter. The recombinant CLuc_SW_ was designed to be produced with FLAG-tag at its carboxyl terminus. Silkworm body fluid was collected from 20 individuals and subjected to ion exchange chromatography using the HiTrap Q HP column (GE Healthcare, IL, United States). The activities of elusion fractions using up to 500 mM NaCl were monitored by light emission activity using synthetic cypridinid luciferin as described previously (Yasuno et al., 2018). The active fractions were pooled and concentrated using a centrifuge filter unit (Amicon Ultra-15, Millipore, MA, United States), followed by desalting using a PD-10 column (GE Healthcare) according to the manufacturer’s protocol. The desalted sample was then further purified using anti-FLAG affinity gel (Merck, Germany).

### Purification of Double Mutant Recombinant Protein_SW_

Dmt CLuc_SW_ was purified by anion exchange chromatography using a MonoQ column (Cytiva, MA, United States). The purification was performed under the conditions of 20 mM HEPES-NaOH pH 7.5, 100–600 mM NaCl in a linear gradient. Finally, gel filtration chromatography using a Superdex 200 Increase column (Cytiva) was carried out under the conditions of 150 mM NaCl and 20 mM HEPES-NaOH pH 7.5 to make the final purified sample. The sample at this stage showed a single band in SDS-PAGE.

### Plasmid Construction for BY-2 Cells and Protein Expression

The Dmt CLuc_BY_ coding sequence was amplified by PCR from chemically synthesized cDNA with a Sma1_cLUC_ F (5′-ggg​ccc​ggg​ATG​AAG​ACT​TTA​ATA​CTC​GCT-3′) and Sal1_cLUC_R (5′-CCC​GTC​GAC​TCA​CTT​GCA​CTC​ATC​TGG​CA-3′) primer pair and cloned into a p35SHSPG vector (Oshima et al., 2011). Then, CaMV35Spro::Dmt CLUC::HSPter was transferred to a pBCKK T-DNA vector (Mitsuda et al., 2006) and transformed into *Rhizobium radiobacter* GV3101.

## Results and Discussion

### Recombinant *Cypridina noctiluca* Luciferase Expression Using Expi293 Expression System

**FIGURE 1:**
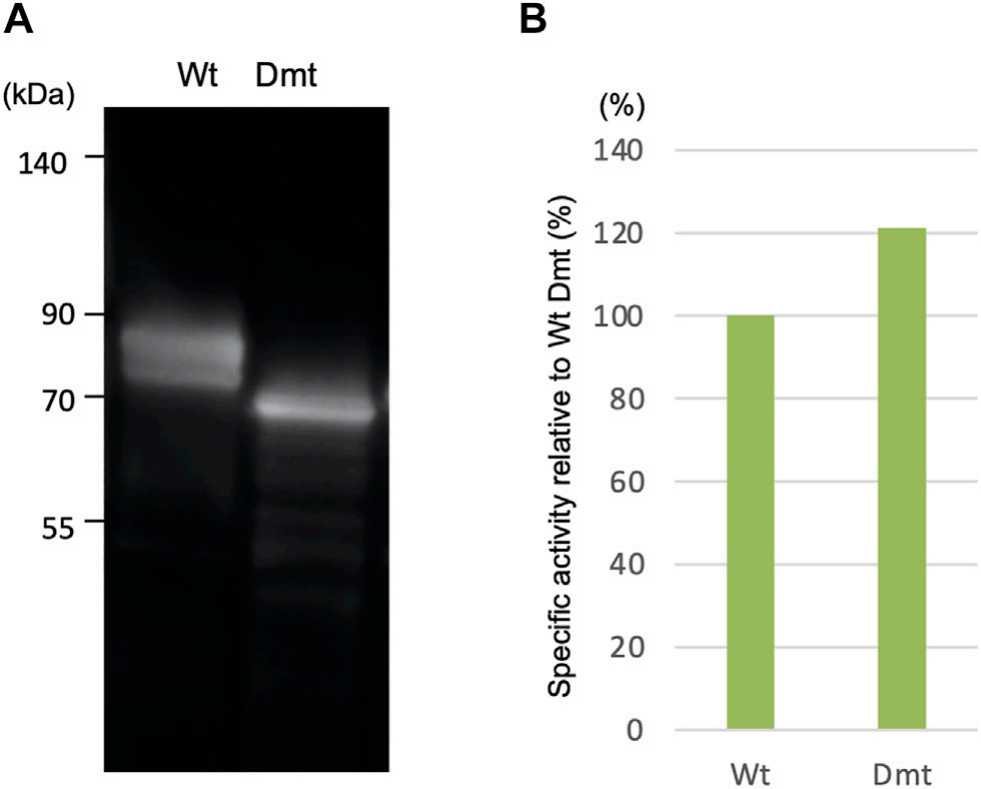
Expression of recombinant wild-type (Wt) and double mutant (Dmt) CLuc_EX_. **(A)** Immunoblot analysis of recombinant proteins using anti-CLuc antibody. **(B)** Relative specific CLuc_EX_ activities in the medium. CLuc_EX_-specific activities were determined by dividing the luminescence intensities by the amount of each protein estimated from immunoblot band intensities. The activity of Wt CLuc_EX_ corresponds to 100%. Note that the immunoblot band looks broad due to glycosylation modification.

In a previous work, we reported the expression of recombinant CLuc deficient in N-glycosylation binding sites (Dmt CLuc_CO_) in a mammalian adherent cell, COS1. In the present study, we first focused on Expi293F cells, which have been adapted to grow in high density and to give higher recombinant protein yields, and tried a suspension culture of the Expi293 expression system, with the expectation of high expression of CLuc. Each expression vector for Wt or Dmt CLuc_EX_ was transfected into Expi293F cells. After 5 days of incubation, the accumulation of CLuc_EX_ in the medium was confirmed by immunoblot analysis using an anti-CLuc antibody (Figure 1A). Mobility shift differences between Wt and Dmt CLuc_EX_ were presumably due to deficiencies in N*-*glycans. Over 90% of the luciferase activities were observed in the medium in both Wt and Dmt CLuc_EX_, indicating that the majority of CLuc produced in Expi293F cells was secreted into the medium efficiently. The activity of CLuc_EX_ was determined by measuring the luminescence intensity using synthetic cypridinid luciferin. The specific activities of Wt and Dmt CLuc_EX_ in the medium were calculated by dividing the luminescence intensities by the estimated intensities of their immunoblot CLuc_EX_ bands (Figure 1B). The visibility of the immunoblot band differed among multiple experiments, and the estimation range was broad. Thus, we performed 3 independent experiments and calculated the average of the specific activity ratio for Dmt CLuc_EX_ relative to that for Wt CLuc_EX_. A typical result is shown in Figure 1B, where the ratio was approximately 120%, but the average ratio was 90.7 ± 21.8%. These data showed that the specific activities of Wt and Dmt CLuc_EX_ were almost the same, unlike the previous result of approximately 20% for Dmt CLuc_CO_ expressed in COS1 cells.

Confirmation of N-glycosylation site mutations and search for O-glycosylation site(s) of Dmt CLuc_EX_ expressed in Expi293F cells by LC-MS.

Dmt CLuc_EX_ protein samples digested with chymotrypsin and/or a mixture of trypsin and Lys-C endopeptidase were analyzed by LC-MS/MS, and the obtained MS/MS spectra (HCD) were searched using Mascot to identify Dmt CLuc_EX_-derived peptides (Supplementary Tables S1–S6). For the trypsin + Lys-C (TL) digest, 81% of peptides were identified, and a peptide containing mutation T184A was included. In addition, for the chymotrypsin digest, 87% of peptides were identified, including a peptide with an N404D mutation (Supplementary Tables S1, S3). These results indicate that both N-glycosylation sites were mutated and lost.

**FIGURE 2:**
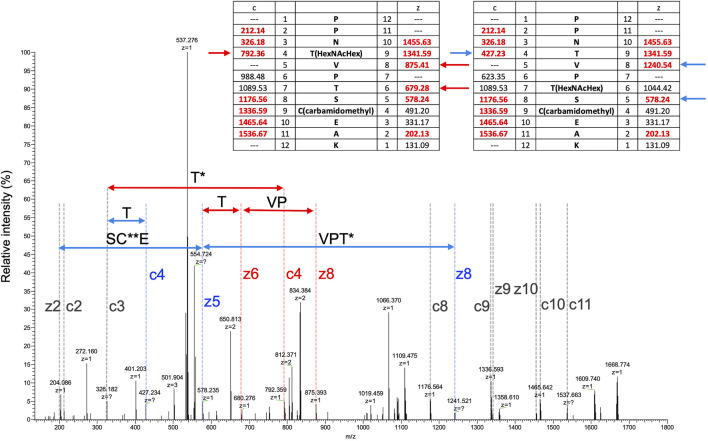
EThcD MS/MS spectrum of a peptide (PPNTVPTSCEAK) having Hex(1) HexNAc(1). MS/MS acquired at the time of (3) in Supplementary Figure S3. Masses of predicted fragment ions are listed in the inset tables. Fragment ion characteristics of glycosylation at Thr-30 are indicated in red, those at Thr-33 are in blue, and common ones are in gray. HexNAcHex and carbamidomethyl modifications are indicated by single and double asterisks, respectively.

To search for other glycosylated site(s), EThcD MS/MS spectra were obtained by triggering with HCD fragments of m/z = 138, 204, or 366, that is, diagnostic fragment ions of glycan corresponding to [HexNAc-CH_6_O_3_]^+^, [HexNAc]^+^, or [HexHexNAc]^+^, respectively. Several O-glycosylated peptides, which were modified with glycan at a serine or threonine residue, were found by the Mascot search, peptide sequences were identified from HCD spectra, and glycosylated sites were assigned by EThcD spectra (Supplementary Tables S2, S6, Supplementary Figures S1–S5). In a TL digest, the HCD MS/MS spectrum of peptide sequence PPNTVPTSCEAK (residues 27–38) having a Hex(1) HexNAc(1) NeuAc(2) modification was found, presenting a series of diagnostic ions of glycan including a NeuAc-H_2_O fragment (m/z = 274) and a ladder-like signal of glycopeptides suggesting a NeuAc-Hex-HexNAc-peptide (Supplementary Figure S1). A partial sequence of the core peptide was assigned. In an acid-treated digest with chymotrypsin + trypsin + Lys-C (CTL), glycopeptides having the same core sequence, PPNTVPTSCEAK, carrying 1 and 2 Hex(1) HexNAc(1) modification(s), were identified. A partial peptide sequence could be assigned from their HCD spectra, but the modification sites were not identified because there are 3 potential Ser/Thr in the peptide (Supplementary Figure S2). Glyco-RIDGE analysis of the acid-treated TL digest revealed a cluster of the core peptide having different glycan compositions, that is, members carrying glycans of Hex:HexNAc:dHex = 0:0:0 (no glycosylation), 0:1:0, 1:1:0, 1:2:0, 2:2:0, and 3:3:0 (data not shown). Extracted ion chromatograms are shown in Supplementary Figure S3 for the signals of 0:0:0, 0:1:0, 1:1:0, and 2:2:0. Signals of 1:2:0 and 3:3:0 were too weak to be seen in the figure. The signal of non-glycosylated peptide was highest even when the scale was reduced to 1/100, suggesting that the glycan occupancy of the peptide was quite low, about 2% of the area. The glycopeptide carrying Hex(1) HexNAc(1) showed twin broad peaks. The EThcD spectra of glycopeptide ions at 3 different retention times were compared (Supplementary Figure S4), revealing multiple O-glycosylated sites in the peptide sequence: Thr-30 and Thr-33 or Ser-34. MS/MS acquired at time (2) in Supplementary Figure S3 suggested that both Thr-30 and Thr-33 were glycosylated (Figure 2). The EThcD spectrum of the peptide having 2 Hex(1) HexNAc(1) was found, and Thr-30 had the Hex(2) HexNAc(2) modification (Supplementary Figure S5). In addition, another sequence EGECIDSSCGTCTR (residues 39–52) was found to have Hex(1) HexNAc(1); however, the site could not be assigned. The rate of O-glycosylation of the peptide was found to be quite low (<1/10^4^) from the area of the extracted ion chromatogram (data not shown).

### Deletion Mutants of O-Glycosylation Residues

**FIGURE 3:**
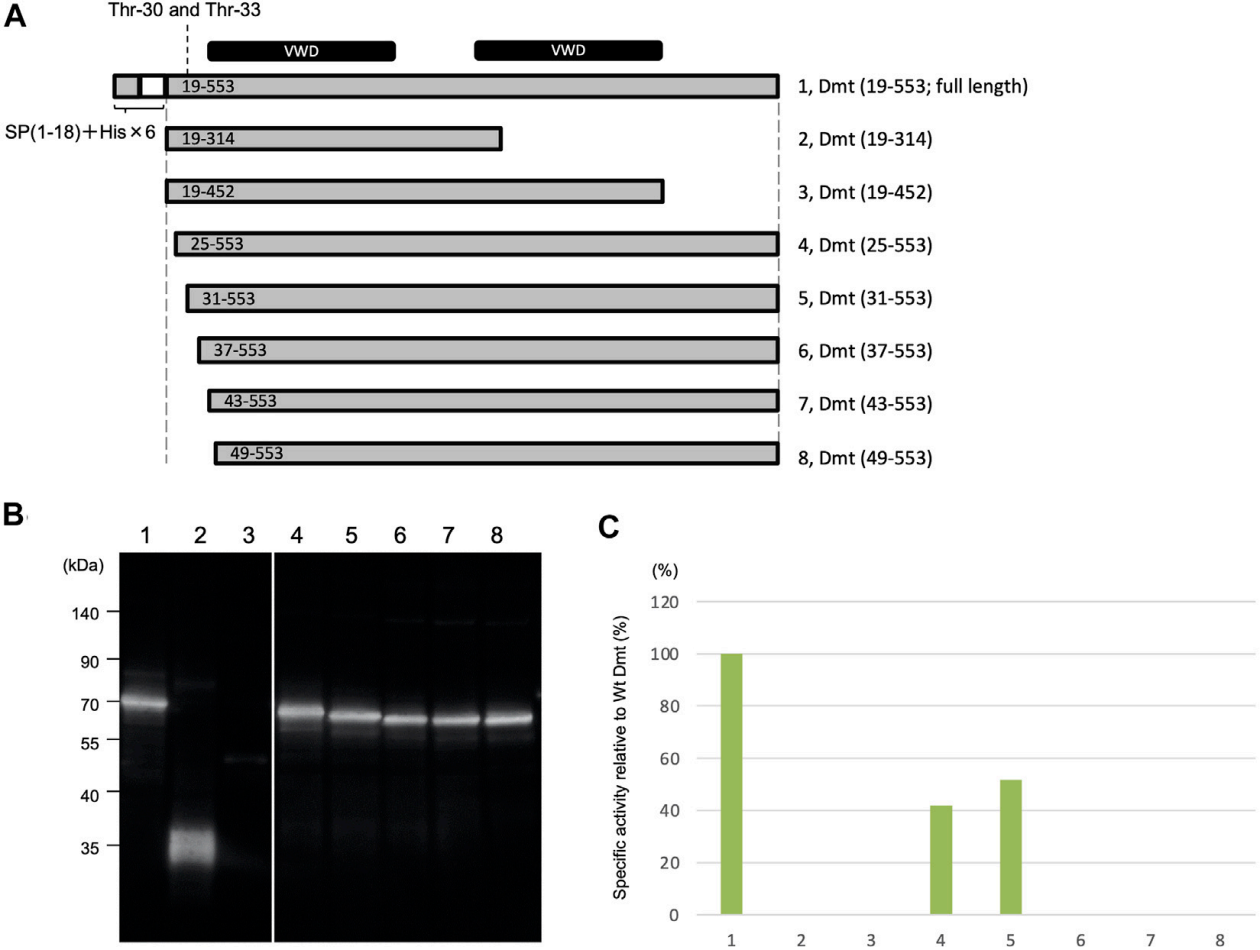
Luminescence activities of Dmt CLuc_EX_ deletion mutants. **(A)** Schematic representation of full-length Dmt CLuc_EX_ and its deletion mutants. Each construct was designed to have a natural signal peptide (SP 1–18) and His tag (His × 6)–TEV sequence at the amino terminus. The O-glycosylation sites are at 30 and 33. VWD-like domains are indicated by black bars at the top of the panel. **(B)** Immunoblot analysis of Dmt CLuc_EX_ deletion mutants. Expi293F cells were transfected with the indicated constructs, and the produced protein secreted into the medium was analyzed by Western blotting using anti-CLuc antibody. **(C)** Relative specific activity of each Dmt CLuc_EX_ deletion mutant. Specific CLuc_EX_ activities were determined as shown in Figure 1. Note that the band in lane 3 is very faint, probably due to the low expression level of this construct.

Deletion mutants were constructed to remove the region around O-glycosylation residues at Thr-30 and Thr-33 (Figure 3A). Protein expression using the Expi293 expression system was confirmed by immunoblot analysis (Figure 3B), and specific activities were determined based on the immunoblot band intensity (Figure 3C). Specific activities were halved in mutants with deletions at residues 19–24 and 19–30 when compared to the full length of Dmt CLuc_EX_. However, no activities were exhibited by the mutants with further deletions at residues 19–36, 19–42, or 19–48, even when the protein amounts were almost equal to those of the full-length protein. These results suggested that the amino acid residues at 31–36 (VPTSCE) were important for CLuc function. This region was highly conserved with the luciferase of the related cypridinid species, including cysteine residues (Supplementary Figure S6).

Moreover, the removal effects of carboxyl-terminal regions of Dmt CLuc_EX_ were investigated. A deletion mutant (19–314) designed to remove the second VWD-like domain exhibited no luciferase activity even though protein production was confirmed at the predicted size of 36 kDa (Figure 3B), suggesting that the residues at 315–553 were indispensable regions for CLuc function. Deletion of the residues at 453–553, which was designed to remove the carboxyl region behind the second VWD-like domain, resulted in a significant reduction of protein production, observed as a slight band around 50 kDa (Figure 3B). Deletion of the region at 453–553 might lead to the instability of the protein or to a reduction of secretion efficiency in this expression system.

### Recombinant *Cypridina noctiluca* Luciferase Expression Using Silkworm

**FIGURE 4:**
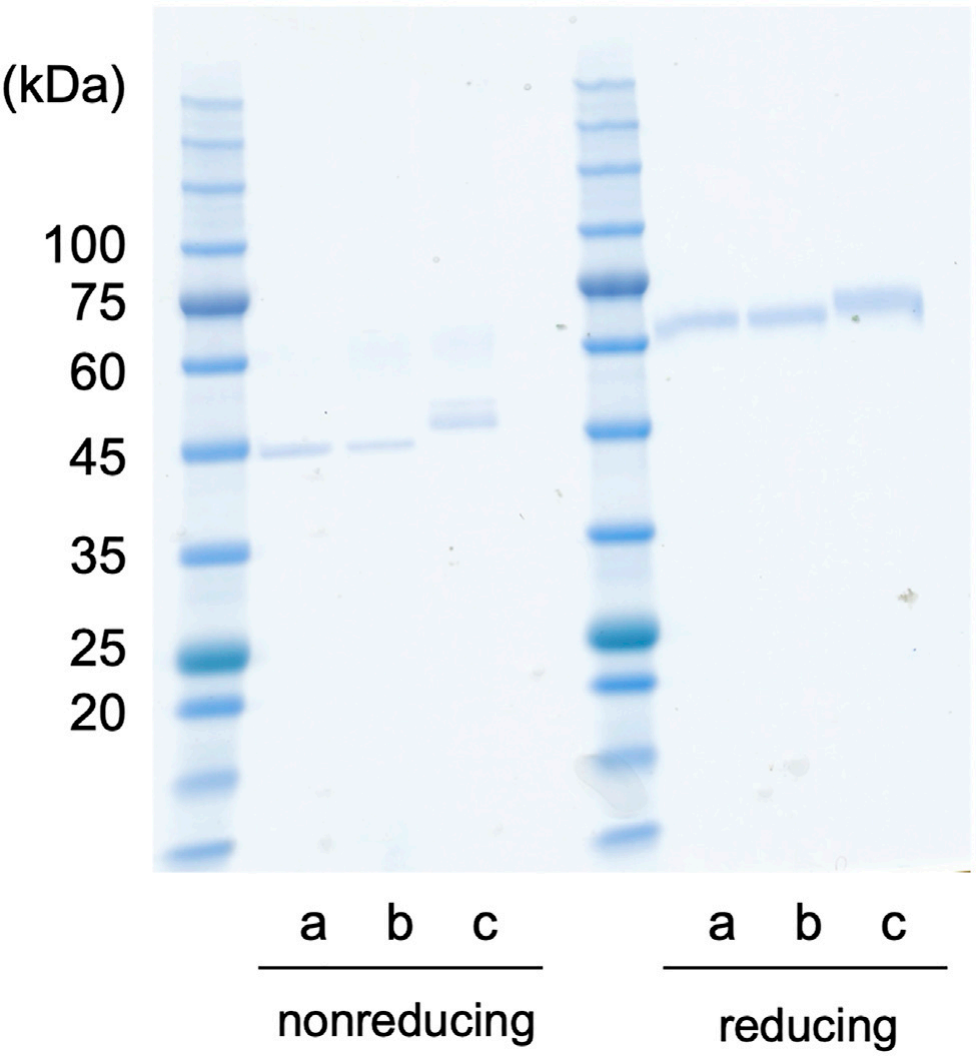
CLuc expression in silkworm. CLuc_SW_ recombinant proteins (200 ng for each lane) were analyzed by SDS-PAGE. The gel mobility was different under reducing or non-reducing conditions. The apparent molecular weight was higher under reducing conditions than under non-reducing ones, showing relative compact folding of the recombinant proteins under non-reducing condition probably due to disulfide bonding. In both cases, the recombinant Wt CLuc_SW_ band looks larger than the Dmt CLuc_SW_ band. (a) Dmt CLuc_SW_. (b) N182D + N404D mutant CLuc_SW_. (c) Wt CLuc_SW_.

The recombinant proteins of Dmt and Wt CLuc_SW_ were produced using the silkworm expression system. First, each recombinant protein was simply purified using anti-FLAG affinity gel, and a single band was observed for each case in the SDS-PAGE analysis (Figure 4). Depending on the protein, treatment with a reducing agent differently affected the mobility in the gel, as shown previously (Mitani et al., 2017). The Wt CLuc_SW_ band looked larger than the Dmt CLuc_SW_ band, and this difference was more obvious under the non-reducing conditions (Figure 4). This size difference would be due to the glycan modification, suggesting that an N-glycan chain would be added to CLuc when produced in silkworm, similar to the case with COS1 and BY-2 cell expressions, as previously reported (Mitani et al., 2017; Yasuno et al., 2018). The luciferase activities of Dmt and Wt CLuc_SW_ were 4.24 × 10^7^ and 1.67 × 10^8^ RLU ng^−1^, respectively. These data indicated that almost 25% activity was retained in the Dmt CLuc_SW_ even without glycan modification. These data were similar to those obtained using a mammalian expression system, which resulted in approximately 20% activity for Wt CLuc_CO_ (Yasuno et al., 2018). We also produced another mutant, N182D + N404D, and expected an improvement in its specific activity. This mutant recombinant protein was successfully produced (Figure 4), but its activity was almost the same as that of Dmt CLuc_SW_ (4.18 × 10^8^ RLU ng^−1^). Thus, we decided to use Dmt CLuc_SW_ for further analysis.

Next, to improve the recombinant protein purity, each sample was successively purified by FLAG-tag affinity chromatography and anion exchange chromatography. The results showed that Dmt CLuc_SW_ was separated into two major peaks (Supplementary Figure S7A). Considering the peak area ratio of the chromatograph, the existence ratio of these fractions was estimated to be about 1:3. The two separated peaks of Dmt CLuc_SW_ were collected, and each sample was subjected to gel filtration chromatography. The higher molecular weight fraction was eluted in the void volume, and the lower one was eluted in the volume corresponding to the molecular weight of the CLuc_SW_ monomer. Next, electrophoresis under native conditions was performed on each of these fractions. As shown in Supplementary Figure S7B, the Wt CLuc_SW_ and the Dmt CLuc_SW_, which were derived from the lower molecular weight fraction in the gel filtration chromatography, showed a single band near the calculated molecular weight in each case, while the Dmt CLuc_SW_, which was obtained as the void fraction in the gel filtration, showed a much higher molecular weight than the monomer. This aggregation is probably due to misfolding, since glycosylation is known to be involved in the correct folding of secreted proteins during translation (Wang et al., 2018). These aggregated Dmt CLuc_SW_ did not show any luciferase activity. These data seemed reasonable because only 25% of Dmt CLuc_SW_ was produced as a monomer, probably with normal activity, while the remaining 75% was aggregated during protein production, resulting in no luciferase activity. Thus, our first estimates of the specific activity of the Dmt CLuc_SW_ when purified only with anti-FLAG affinity were artificially low due to the presence of a high percentage of aggregated, non-functional luciferase. In the case of Dmt CLuc_CO_ expressed using COS1 cells, the specific activity was 20% of that of the Wt CLuc_CO_ (Yasuno et al., 2018). Although we did not perform further purification for this protein due to the limited amount of protein produced, 80% of the recombinant protein may have aggregated during protein production, as in the case of the silkworm expression system.

### Recombinant *Cypridina noctiluca* Luciferase Expression Using Suspension-Cultured Tobacco BY-2 Cells

Wt CLuc_BY_ was efficiently expressed using cultured tobacco BY-2 cells (Mitani et al., 2017), and thus, we tried to produce Dmt CLuc_BY_. However, none of the clones produced recombinant Dmt CLuc_BY_ even though we tested 4 independent BY-2 callus clones. This result suggested that the N-glycosylation of CLuc_BY_ was essential for its recombinant protein production in BY-2 cells.

## Conclusion

In this study, we produced recombinant mutant CLuc lacking N-glycosylation motifs using Expi293F cells, silkworms, and tobacco BY-2 cells. The relative activity of the produced Dmt CLuc_EX_ was almost the same as that of the Wt CLuc_EX_, whereas Dmt CLuc_CO_ was approximately 20% of that of Wt CLuc_CO_ in the previous study (Yasuno et al., 2018). In the case of the silkworm system, the relative activity of mutant CLuc purified using only anti-FLAG affinity gel was 25% of that of Wt CLuc_SW_. However, further purification revealed that approximately 75% of the FLAG-purified recombinant protein was aggregated and its enzyme activity was lost. In the BY-2 cells, our attempt to express Dmt CLuc_BY_ resulted in no recombinant protein production despite the use of 4 independent transgenic callus clones. Taken together, our results suggested that the productivity of CLuc mutated in the N-glycosylation sites differed depending on the expression host. Therefore, we should choose the recombinant protein expression system carefully in order to minimize the effect of glycosylation, especially for cases such as protein crystallization, which requires the removal of glycan. In our case, the Expi293 expression system was the best choice for Dmt CLuc production. To understand the reason for these differences in protein production efficiency, we need to study the protein production pathway in detail and to investigate which point is critical for the efficient production of recombinant protein. Finally, we identified important residues involved in CLuc activity. Crystallographic analysis will be necessary to clarify the functions of these residues in the oxidation process of cypridinid luciferin.

## Data Availability

The datasets presented in this study can be found in online repositories. The names of the repository/repositories and accession number(s) can be found below: https://repository.jpostdb.org/ and JPST001397, PXD029916.
