# Supplementary material for: Host-Dependent Producibility of Recombinant Cypridina noctiluca Luciferase With Glycosylation Defects
Source: Front Bioeng Biotechnol. 2022 Feb 7;10:774786. doi: 10.3389/fbioe.2022.774786 (PMC8859458; doi:10.3389/fbioe.2022.774786)
Supplement: Supplementary file 1 [file DataSheet1.PDF]

Supplementary Material

Host dependent producibility of recombinant *Cypridina noctiluca* luciferase with glycosylation defects.

Supplemental Figures

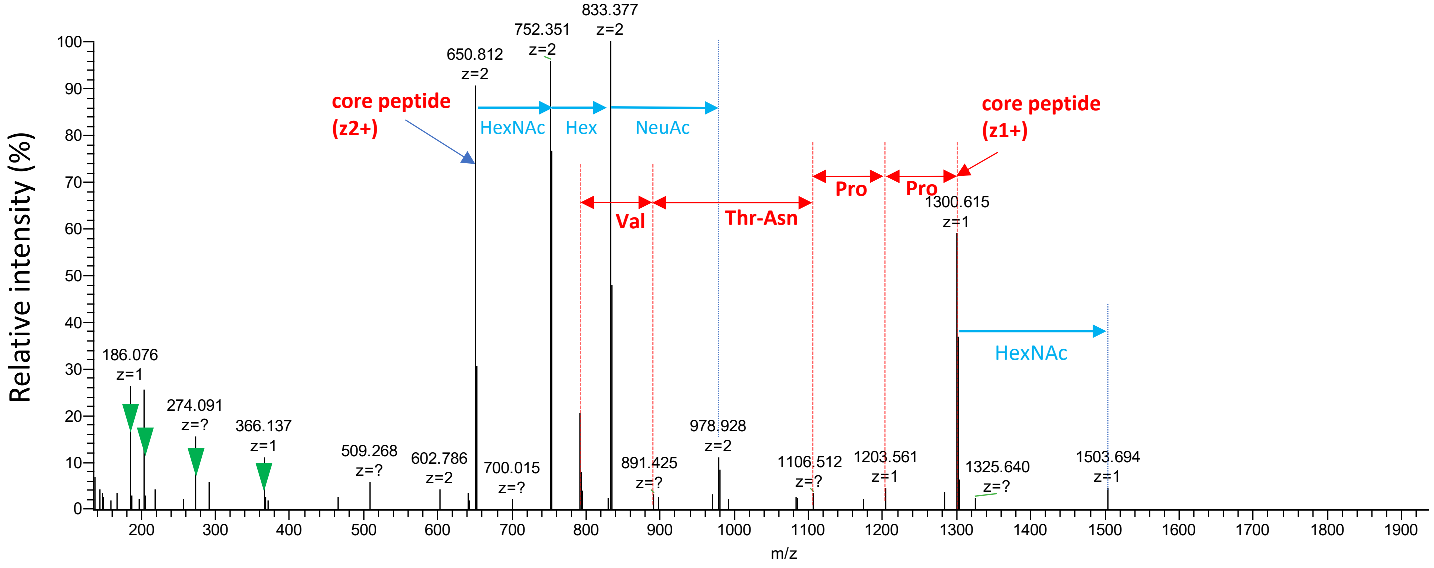

Supplementary Figure 1. HCD MS/MS spectrum of a peptide PPNTVPTSCEAK having Hex(1)HexNAc(1)NeuAc(2) for Trypsin+Lys-C digest of Dmt CLuc$_{EX}$. Precursor ion: m/z=1124.4752 (z=2+). Fragment ions of m/z 186, 204, 274, and 366 indicated with green arrowheads are a series of diagnostic ions of glycan corresponding to [HexNAc-H_2O]$^+$, [HexNAc]$^+$, [NeuAc-H_2O]$^+$, and [HexHexNAc]$^+$, respectively. Ladder-like signals of core peptide and glycopeptides fragmented in part are observed, suggesting O-glycosylation of the core peptide. Partial amino acid sequence of the core peptide could be assigned as red line.

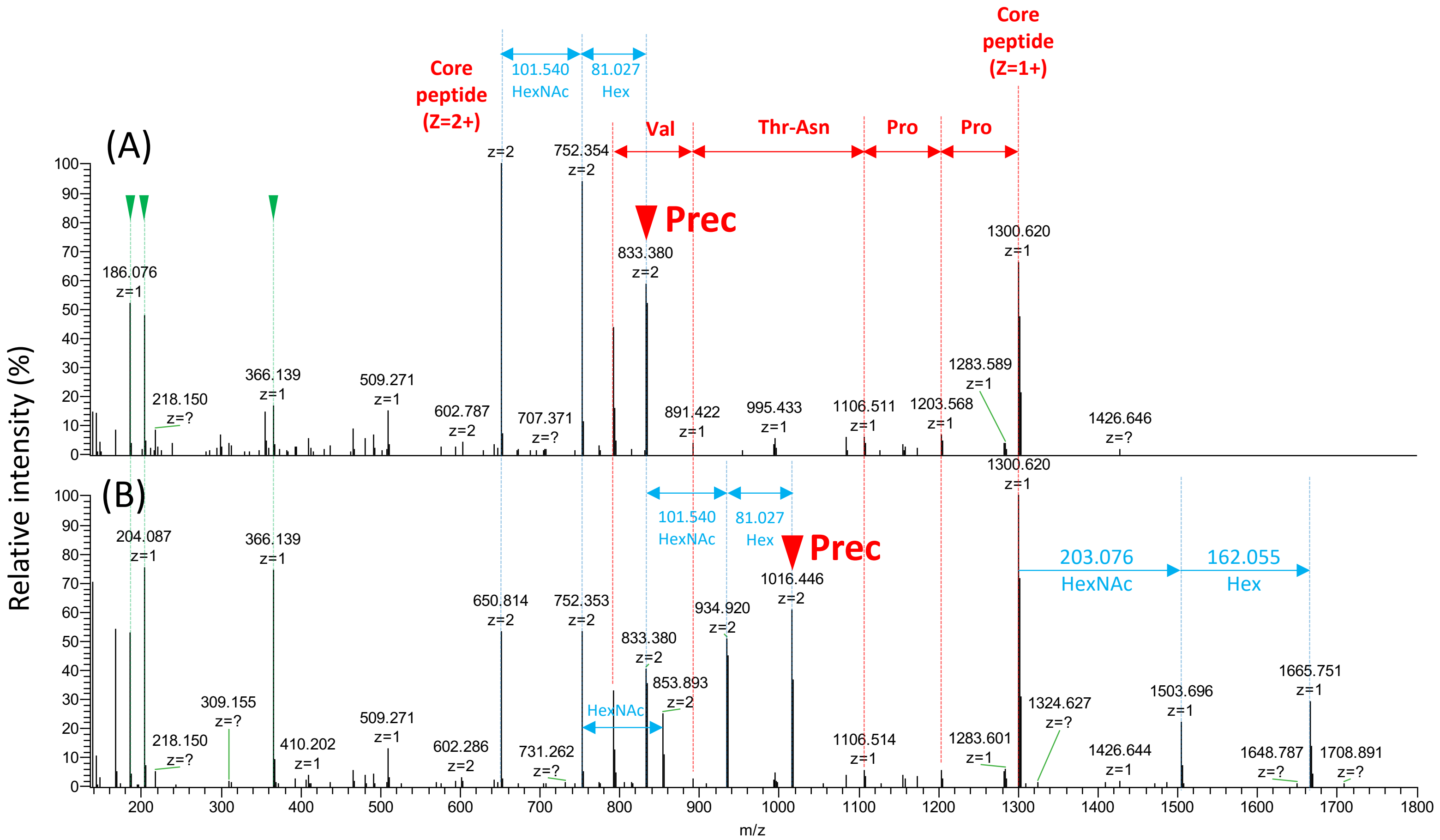

Supplementary Figure 2. HCD MS/MS spectra of a peptide PPNTVPTSCEAK having 1 (A) and 2 (B) Hex(1)HexNAc(1) for an acid-treated Chymotrypsin+Trypsin+Lys-C digest of Dmt $CLuc_{EX}$. Precursor ions are indicated with red arrowhead (Prec). Fragment ions of m/z 186, 204, and 366 indicated with green arrowheads are a series of diagnostic ions of glycan corresponding to $[HexNAc\text{-}H_2O]^+$, $[HexNAc]^+$, and $[HexHexNAc]^+$, respectively. Ladder-like signals of core peptide and glycopeptides fragmented in part are observed, suggesting O-glycosylation of the core peptide. Fragment ions indicated with red dotted line suggest the partial sequence of a common core peptide, PP(NT)V.

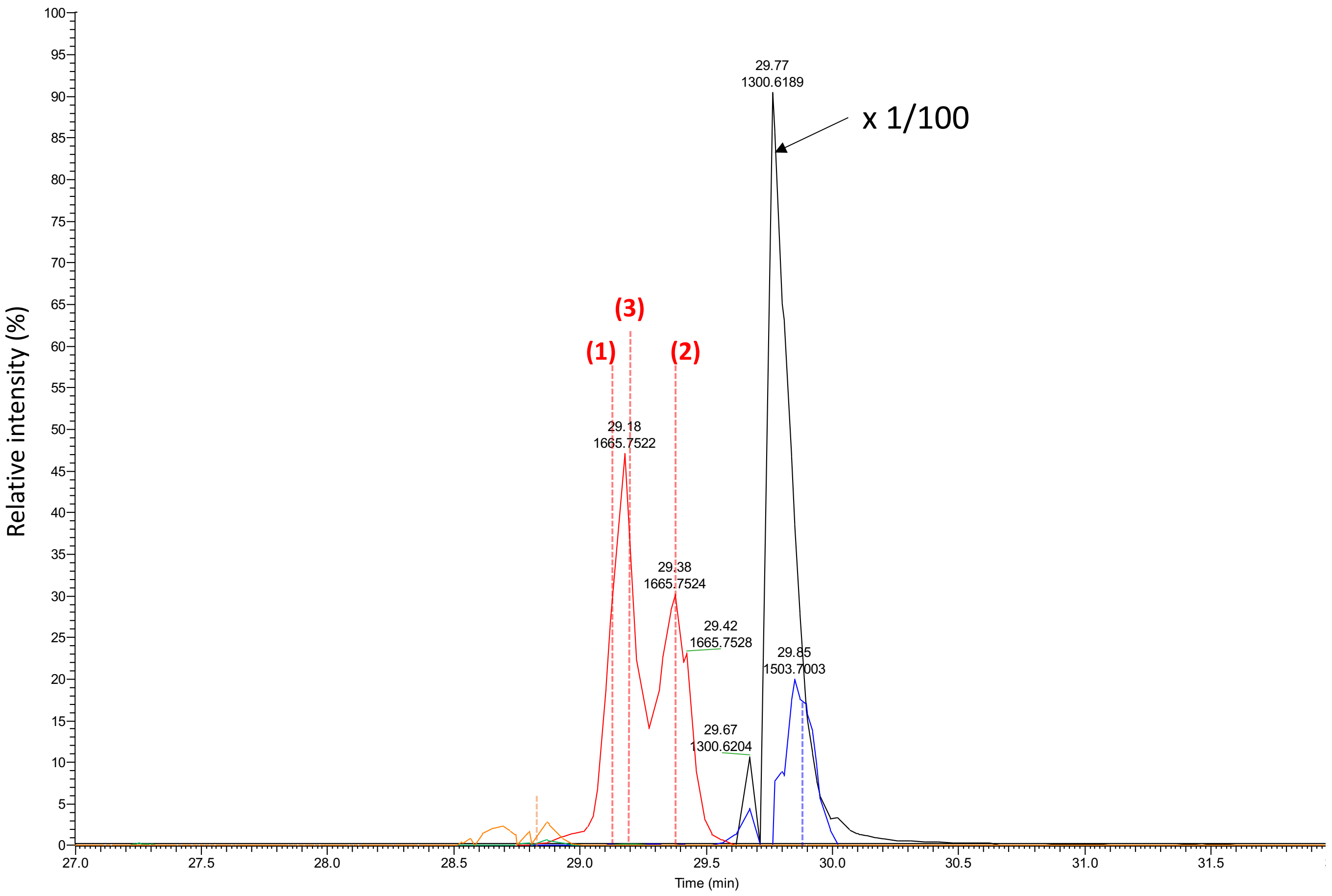

Supplementary Figure 3. Extracted ion chromatograms of peptide PPNTVPTSCEAK carrying a different glycan. Black: not glycosylated (scale is reduced to 1/100) , blue: peptide + HexNAc(1), red: peptide + Hex(1)HexNAc(1), orange: peptide + Hex(2)HexNAc(2). MS/MS spectra acquired at the time indicated with vertical line are presented in Supplementary Figure 4. Full scale of relative intensity: 1.6 x 10^7.

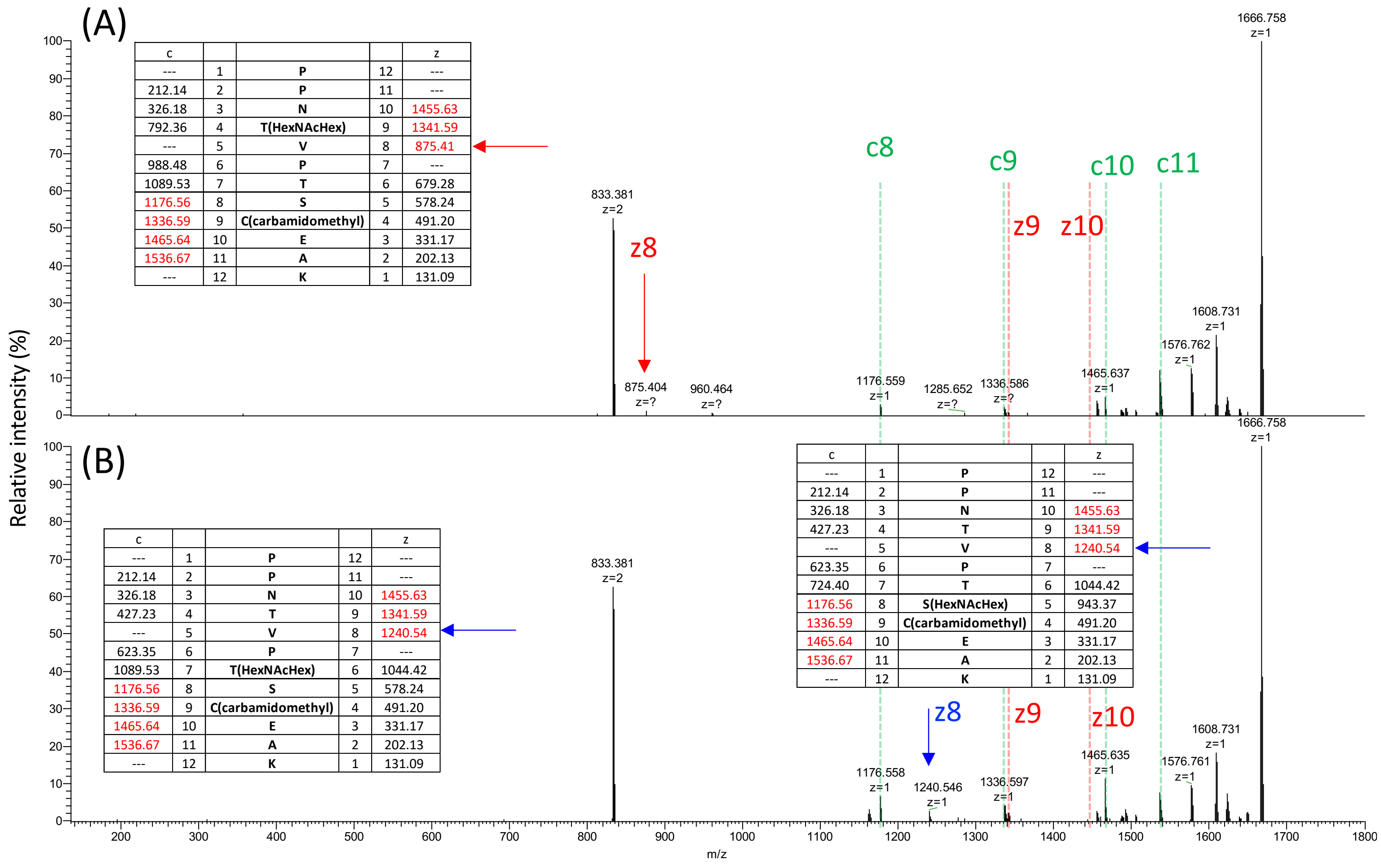

(A)

c				z
---	1	P	12	---
212.14	2	P	11	---
326.18	3	N	10	1455.63
792.36	4	T(HexNAcHex)	9	1341.59
---	5	V	8	875.41
988.48	6	P	7	---
1089.53	7	T	6	679.28
1176.56	8	S	5	578.24
1336.59	9	C(carbamidomethyl)	4	491.20
1465.64	10	E	3	331.17
1536.67	11	A	2	202.13
---	12	K	1	131.09

(B)

c				z
---	1	P	12	---
212.14	2	P	11	---
326.18	3	N	10	1455.63
427.23	4	T	9	1341.59
---	5	V	8	1240.54
623.35	6	P	7	---
1089.53	7	T(HexNAcHex)	6	1044.42
1176.56	8	S	5	578.24
1336.59	9	C(carbamidomethyl)	4	491.20
1465.64	10	E	3	331.17
1536.67	11	A	2	202.13
---	12	K	1	131.09

c				z
---	1	P	12	---
212.14	2	P	11	---
326.18	3	N	10	1455.63
427.23	4	T	9	1341.59
---	5	V	8	1240.54
623.35	6	P	7	---
724.40	7	T	6	1044.42
1176.56	8	S(HexNAcHex)	5	943.37
1336.59	9	C(carbamidomethyl)	4	491.20
1465.64	10	E	3	331.17
1536.67	11	A	2	202.13
---	12	K	1	131.09

Supplementary Figure 4. EThcD MS/MS spectra of the peptide (PPNTVPTSCEAK) having Hex(1)HexNAc(1). (A) MS/MS acquired at the time of (1) in Supplementary Figure 3. (B) MS2 acquired at the time of (2). Masses of predicted fragment ions are listed in the inset tables. Fragment ions of c8 are suggesting the glycosylated site of Thr-30 (A) and Thr-33 or Ser-34 (B), respectively.

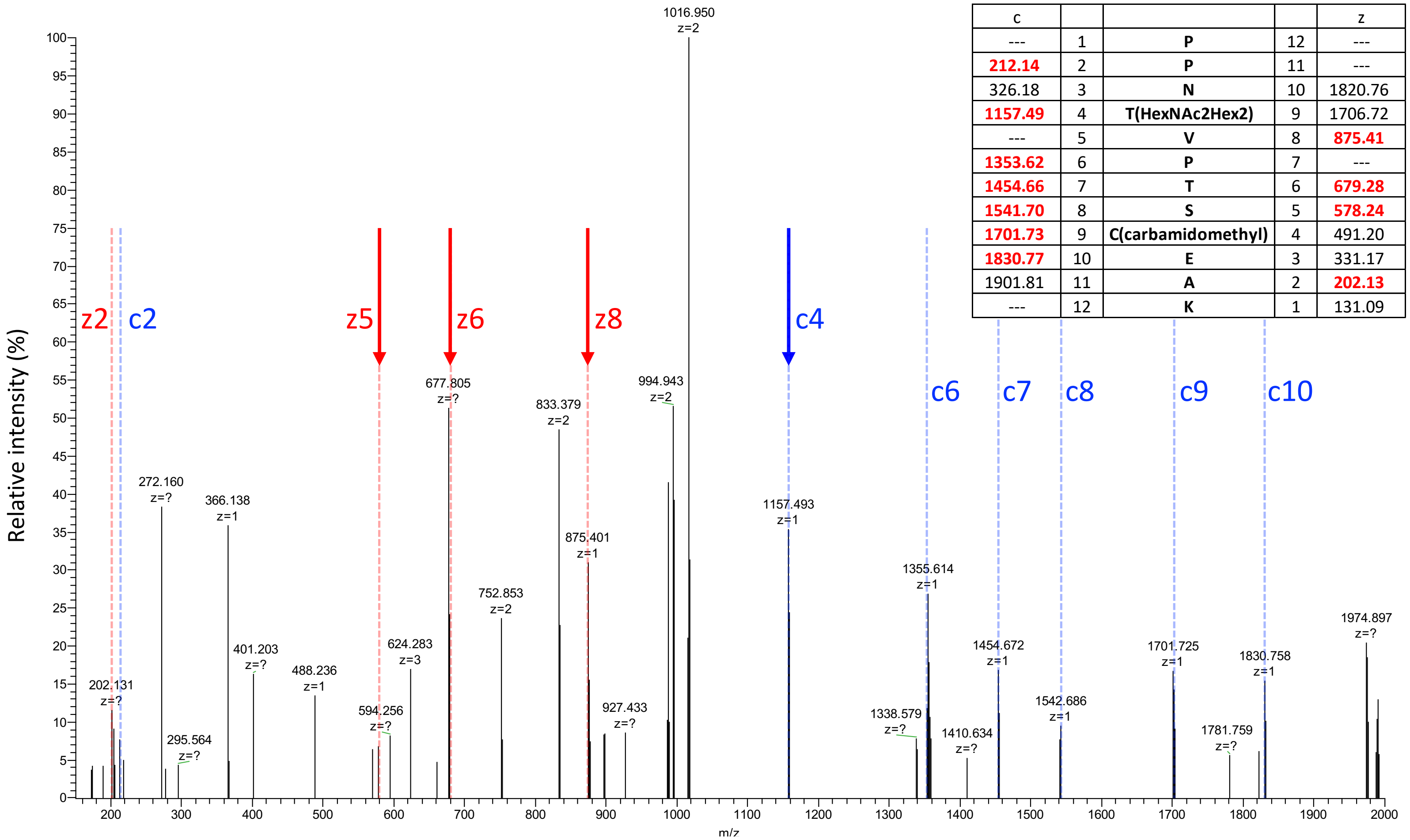

c				z
---	1	**P**	12	---
**212.14**	2	**P**	11	---
326.18	3	**N**	10	1820.76
**1157.49**	4	**T(HexNAc2Hex2)**	9	1706.72
---	5	**V**	8	**875.41**
**1353.62**	6	**P**	7	---
**1454.66**	7	**T**	6	**679.28**
**1541.70**	8	**S**	5	**578.24**
**1701.73**	9	**C(carbamidomethyl)**	4	491.20
**1830.77**	10	**E**	3	331.17
1901.81	11	**A**	2	**202.13**
---	12	**K**	1	131.09

Supplementary Figure 5. EThcD MS/MS spectrum of the peptide (PPNTVPTSCEAK) having Hex(2)HexNAc(2). Masses of predicted fragment ions are listed in the inset table. Fragment ions suggesting glycosylation at Thr-30 are indicated in red and blue arrows.

```
QOS14273.1_Vargula_tsujii            --------------------------------------------------
QOS14271.1_Maristella_sp             MRFPSIFTAVLFAASSALAAPVNTTTEDETAQIPAEAVIGYSDLEGDFDV
QOS14272.1_Kornickeria_basting       MRFPSIFTAVLFAASSALAAPVNTTTEDETAQIPAEAVIGYSDLEGDFDV
AAA30332.1_Vargula_hilgendorfi       ------MKIIILSVILAYCVTDN---------------------------
BBG57195.1_Cypridina_noctiluca       ------MKTLILAVALVYCVTVN---------------------------

QOS14273.1_Vargula_tsujii            --------------------------------MWLQNLFILAVGICFCAA
QOS14271.1_Maristella_sp             AVLPFSNSTNNGLLFINTTIASIAAKEEGVSLEKREAQDCYEFTLEKREA
QOS14272.1_Kornickeria_basting       AVLPFSNSTNNGLLFINTTIASIAAKEEG-------------VSLEKREA
AAA30332.1_Vargula_hilgendorfi       ---------------------------------------------CQDAC
BBG57195.1_Cypridina_noctiluca       ---------------------------------------------CQECP

QOS14273.1_Vargula_tsujii            QDCYESTWASNDYPSSCEALNGRCVDSACGSCDEVLFGDGLCENAGGASP
QOS14271.1_Maristella_sp             QDCYEFTWASNDFPSSCEAINGRCVDSACGSCDEVLFGDGLCENAGGASP
QOS14272.1_Kornickeria_basting       KDCFESSFHSLEFPSSCEAQNGICIDSECKDCNEVMFGDGLCENAGGASP
AAA30332.1_Vargula_hilgendorfi       PVEAEPPSSTPTVPTSCEAKEGECIDTRCATCKRDILSDGLCENKPGK--
BBG57195.1_Cypridina_noctiluca       YVADPPN----TVPTSCFAKEGECIDSSCSTCTRDILSDGLCENKPGK--
                                                 *:**** :* *:*: *  * . ::.******  *

QOS14273.1_Vargula_tsujii            KCCRDCPEIVRCRASAAGYFHTFYGQRFNLQVPGTYLLSEDCVGGLWSLY
QOS14271.1_Maristella_sp             KCCRDCPEIVRCRASAAGFFHTFYGQRFNLQVPGTYLLSEDCVGGLWSLY
QOS14272.1_Kornickeria_basting       KCCRDCPEIVRCRASAAGFFTTFYGKKFNLQEPGTYLLSEDCVGGLWSLY
AAA30332.1_Vargula_hilgendorfi       TCCRMCQYVIECRVEAAGYFRTFYGKRFNFQEPGKYVLARGTKGGDWSVT
BBG57195.1_Cypridina_noctiluca       TCCRMCQYVIECRVEAAGWFRTFYGKRFQFQEPGTYVLGQGTKGGDWKVS
                                     .*** *  ::.**..***:* ****::*::* **.*:*...  ** *.:

QOS14273.1_Vargula_tsujii            VNLANIEGEKGSVLDSVKMVVGDVTVDIKQKVGD-VTVNGGSVEIDSNPF
QOS14271.1_Maristella_sp             VNLANIEGEKGAVLDSVKMVVGDVTLDIKQKVGS-ITVNGGSVEIDSNPF
QOS14272.1_Kornickeria_basting       VTLVNIAGEKGAVLGSVKMIVGEVTVDIVQKGGP-VTVNGGSVAIDSNPF
AAA30332.1_Vargula_hilgendorfi       LTMENLDGQKGAVLTKTTLEVAGDVIDITQATADPITVNGGADPVIANPF
BBG57195.1_Cypridina_noctiluca       ITLENLDGTKGAVLTKTRLEVAGDIIDIAQATENPITVNGGADPIIANPY
                                     :.: *: * **:** .. : *.   :** *     :*****:  : :**:

QOS14273.1_Vargula_tsujii            SIGDVTIAIVHTPNFDVSVIEFLKLVTFDILQGRAFRLAPDFLYADRTCG
QOS14271.1_Maristella_sp             SIGDVTIAVVYTPYFSVSVIEFLKLVTFDILQGRAFRLAPDFLYADRTCG
QOS14272.1_Kornickeria_basting       SIGDVTIAIVHTPNFDVAVIEFLKLVTFDIIHGRAFRLAPDFLYRDRTCG
AAA30332.1_Vargula_hilgendorfi       TIGEVTIAVVEIPGFNITVIEFFKLIVIDILGGRSVRIAPDTANKGLISG
BBG57195.1_Cypridina_noctiluca       TIGEVTIAVVELPGYNITVIEFFKLIVIDILGGRSVRIAPDTANKGMISG
                                     :**:****:*  * :.::****:**:.:**: **:.*:***    .  .*

QOS14273.1_Vargula_tsujii            LCG-VMSDKPTDFIDNPDQLAIQDQMNQDVDGCPLSGNPSDVEYCLNKMQ
QOS14271.1_Maristella_sp             LCG-LMSDEPSDFIDDPDQLAIQDIMNQDVEGCPLSGNPSDAEYCLNKMQ
QOS14272.1_Kornickeria_basting       LCG-VMSNEPTDFIDNPDQLAVQDKINKDIDGCPLSGNPSDVEYCKNKMQ
AAA30332.1_Vargula_hilgendorfi       ICGNLEMNDADDFTTDADQLAIQPNINKEFDGCPFYGNPSDIEYCKGLME
BBG57195.1_Cypridina_noctiluca       LCGDLKMMEDTDFSSDPEQLAIQPKINQEFDGCPLYGNPEDITYCKGLLE
                                     :** :   .  **  :.:***:*  :*::.:***: ***.*  ** . ::

QOS14273.1_Vargula_tsujii            PYKDGCVNKNDVHFGTYLYACALAYCMGGDDRVEDVIFEYAEACVEPIGR
QOS14271.1_Maristella_sp             PYKDGCINKNDVHFGTYLYACALAYCMGGDDRVEDVIFEYAEACVEPIGR
QOS14272.1_Kornickeria_basting       PYKVACINYNDVNFATYLYSCALAYCMGGEDRVEDVIFDYVEACVEPITR
AAA30332.1_Vargula_hilgendorfi       PYRAVCRN--NINFYYYTLSCAFAYCMGGEERAKHVLFDYVETCAAPETR
BBG57195.1_Cypridina_noctiluca       PYKDSCRN--NINFYYYTLSCAFARCMGGDERASHVVLDYRETCAAPETR
                                     **:  * *  :::*  *  :**:* ****::*...*:::* *:*. *  *

QOS14273.1_Vargula_tsujii            ATCVMNGHTYYDTFDKTSYQFQAPC-KVLFAKDCTDDDWEVTITHKAVGE
QOS14271.1_Maristella_sp             ATCVMNGHTYYDTFDKSSYQFQAPC-KVLFAKDCTDDEWEVTITHKEAGE
QOS14272.1_Kornickeria_basting       ATCVMNGHTYYDTFDKTSYQFQAPC-KVLFAKDCAGDEWEVTITHKAAGT
AAA30332.1_Vargula_hilgendorfi       GTCVLSGHTFYDTFDKARYQFQGPCKEILMAADCYWNTWDVKVSHRDVES
BBG57195.1_Cypridina_noctiluca       GTCVLSGHTFYDTFDKARYQFQGPCKEILMAADCYWNTWDVKVSHRNVDS
                                     .***:.***:******: ****.** ::*:* **  : *:*.::*: .

QOS14273.1_Vargula_tsujii            YTEVEKVTVRYFQTLIDLISETKKVFVNGSEVSVPYNYGDTSIYMYDN-L
QOS14271.1_Maristella_sp             YTEVEKVTVRYFQTLIDLVAENKKVFVNGTEVSVPYNYGDTSIYMYDN-L
QOS14272.1_Kornickeria_basting       YTEVEKVTVRYFQTLIDLISEGKQVLVNGTEVSVPYNKGDTSIYMYDN-L
AAA30332.1_Vargula_hilgendorfi       YTEVEKVTIRKQSTVVDLIVDGKQVKVGGVDVSIPYSSENTSIYWQDGDI
BBG57195.1_Cypridina_noctiluca       YTEVEKVRIRKQSTVVELIVDGKQILVGGEAVSIPYSSQNTSIYWQDGDI
                                     ******* :*  .*:::*: : *:: *.*  **:**.  :****  *. :

QOS14273.1_Vargula_tsujii            ITTAVLPGAVVVKYNFEQMLALHIRDPYEADSCGLCGIWDLDKSNEGPDT
QOS14271.1_Maristella_sp             VTTAVLPGAVVVKYNFEQMLALHIRDPYEADSCGLCGIWDLDKSNDGPDA
QOS14272.1_Kornickeria_basting       ITTAVLPGAVVVKYNFEQMLALHIRDPYERRSCGLCGIWDLDKSNDGPDN
AAA30332.1_Vargula_hilgendorfi       LTTAILPEALVVKFNFKQLLVVHIRDPFDGKTCGICGNYNQDSTDDFFDA
BBG57195.1_Cypridina_noctiluca       LTTAILPEALVVKFNFKQLLVVHIRDPFDGKTCGICGNYNQDFSDDSFDA
                                     :***:** *:***:**:*:*.:*****::  :**:** :: * :::  *

QOS14273.1_Vargula_tsujii            KYVDCEPTPNPPTCTADKEAEARELCQNMFPAS--LDDQCNICYKADRVE
QOS14271.1_Maristella_sp             QHADCEPTPNPPTCTADKEAEARELCQNMFPAS--IDDKCNICYKADRVE
QOS14272.1_Kornickeria_basting       QYVDCEPTPNPATCTADQEAEARELCQNMFPAS--LDDECDICYKSDRVE
AAA30332.1_Vargula_hilgendorfi       EG-ACALTPNPPGCTEEQKPEAERLCNNLFDSS--IDEKCNVCYKPDRIA
BBG57195.1_Cypridina_noctiluca       EG-ACDLTPNPPGCTEEQRPEAERLCNSLFVGQSDLDQKCNVCYKPDRVE
                                     :   *  ****. ** ::..**..**:.:* ..  :*::*::***.**:

QOS14273.1_Vargula_tsujii            RCMYEYCLGGLEGFCQHAGTVIDECFVRHGDDLQYPPQCK----------
QOS14271.1_Maristella_sp             RCMYEYCLGGLEAFCQHAGTVIDECFVRHGDDLQYPPQCKAAAS-FLEQK
QOS14272.1_Kornickeria_basting       RCMYEYCLGGMDYFCKHAGTVIDECFVRHGDDLQLPPQCTKAAASFLEQK
AAA30332.1_Vargula_hilgendorfi       RCMYEYCLRGQQGFCDHAWEFKKECYIKHGDTLEVPPECQ----------
BBG57195.1_Cypridina_noctiluca       RCMYEYCLRGQQGFCDHAWEFKKECYIKHGDTLEVPDECK----------
                                     ******** * : **.**  . .**:::*** *: * :*

QOS14273.1_Vargula_tsujii            -----------------  48%
QOS14271.1_Maristella_sp             LISEEDLNSAVDHHHHHH  49%
QOS14272.1_Kornickeria_basting       LISEEDLNSAVDHHHHHH  50%
AAA30332.1_Vargula_hilgendorfi       -----------------  84%
BBG57195.1_Cypridina_noctiluca       -----------------
```

Supplementary Figure 6. Multiple alignment of cypridinid luciferase. Conserved cysteine residues and Potential O-glycan modification residues are highlighted with green and cyan background, respectively. Functionally critical amino acid residues 31-36 are indicated in a red box. Underline, predicted signal peptide; bold italic, VWD-like domain predicted by blastP. Percentage of identical amino acids to *Cypridina noctiluca* obtained by BlastX is indicated at the last of each sequence.

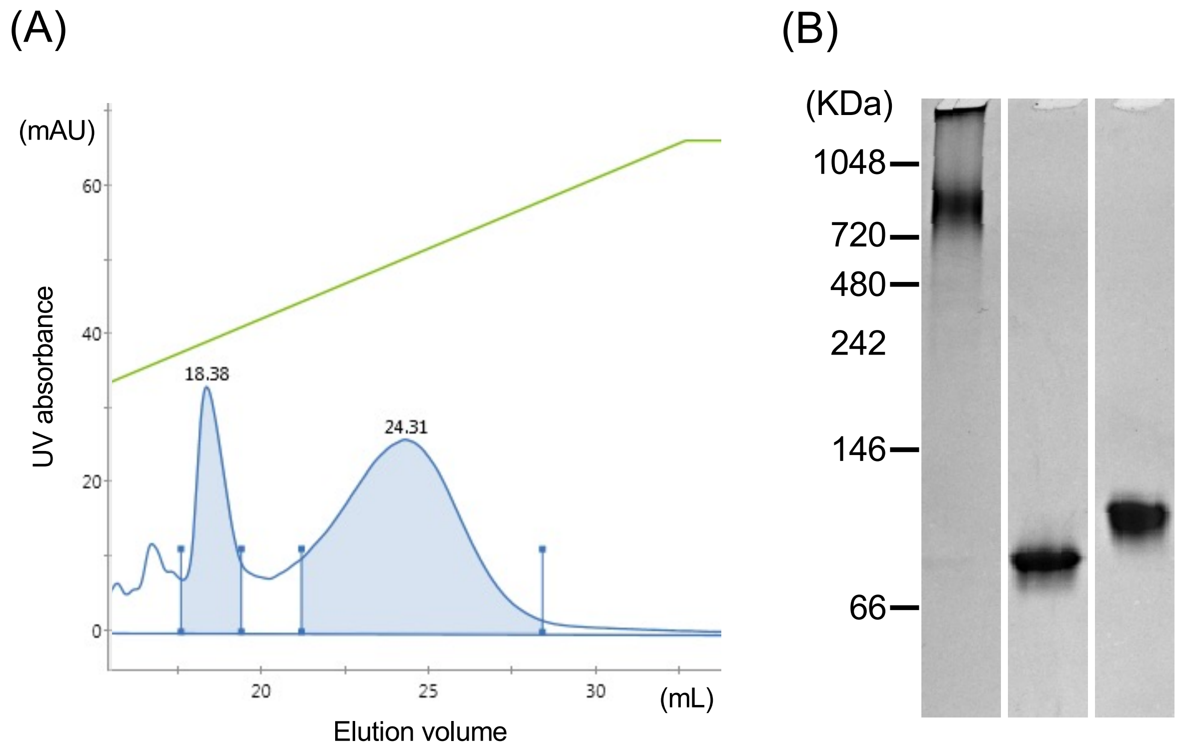

Supplementary Figure 7. The purification results of recombinant CLuc expressed using the silkworm system. (A) Chromatogram of Anion Exchange Chromatography of Recombinant Dmt $CLuc_{SW}$. The two major peaks are indicated in blue at 18.38 and 24.31 mL elution peaks, respectively. (B) Native PAGE results of $CLuc_{SW}$ recombinant proteins. Left lane, Dmt $CLuc_{SW}$ fraction with the peak at 24.31 mL; middle lane, Dmt $CLuc_{SW}$ fraction with the peak at 18.38 mL. For the molecular size comparison, Wt $CLuc_{BY}$ is shown in the right lane.
